# Conventional Salvage Chemotherapy Without Stem Cell Rescue for Relapsed or Refractory Extracranial Germ Cell Tumors: Insights From the Tertiary Cancer Center in a Developing Country

**DOI:** 10.7759/cureus.97904

**Published:** 2025-11-26

**Authors:** Tehreem Zahra, Maryam Jamil, Muhammad Shahid, Niaz Ali, Urooj Fatima, Najma Shaheen

**Affiliations:** 1 Pediatric Oncology, Shaukat Khanum Memorial Cancer Hospital and Research Centre, Lahore, PAK; 2 Pharmacology and Therapeutics, Shaukat Khanum Memorial Cancer Hospital and Research Centre, Lahore, PAK

**Keywords:** 1.event free survival (efs, 2.overall survival (os), 3.salvage chemotherapy(sc), 4.high risk (hr), 5.intermediate risk(ir), germ cell tumor(gct), low risk (lr), relapsed-refractory(rel/ref)

## Abstract

Background

Evidence regarding outcomes and prognostic determinants of extracranial relapsed or refractory germ cell tumors (GCTs) in children remains scarce. The present study evaluates remission rates and survival outcomes of pediatric relapsed or refractory GCTs managed with conventional salvage chemotherapy regimens, without the use of stem cell rescue, at a single tertiary cancer centre in a developing country.

Methods

This retrospective cohort study was conducted at Shaukat Khanum Memorial Cancer Hospital and Research Centre, Lahore, Pakistan, from January 2011 to December 2022. Patients aged ≤18 years with histologically confirmed extracranial GCTs who received definitive treatment, or presented with relapsed/progressive disease after prior chemotherapy, were included. Demographic, clinical, treatment, and outcome data were extracted from electronic records. Event-free survival (EFS) and overall survival (OS) were calculated from initiation of salvage chemotherapy.

Results

Out of 283 patients treated, 51 (18%) with relapsed (n=41, 14.5%) or refractory (n=10, 3.5%) extracranial GCTs were analyzed. The cohort included 25 males (49%) and 26 females (51%), with a median age of 8 years (IQR: 3.0-12.0). Primary tumors were gonadal in 33 (64.7%) and extragonadal in 18 (35.3%) patients. Yolk sac histology was most frequent (n=27, 52.9%), and 24 (47.1%) patients had high-risk disease. Vinblastine, ifosfamide and cisplatin/vinblastine, ifosfamide, carboplatin (VeIP/VeIC) was the commonest salvage regimen (54.9%). At median follow-up of 32 months, 26 (51.0%) were alive without disease, and 14 (27.5%) had died. Five-year OS and EFS were 72.5% and 50%. High-risk disease independently predicted higher mortality (HR: 12.27, 95% CI: 1.51-99.6; p=0.019).

Conclusions

Salvage chemotherapy regimens provide an effective option for children with relapsed or refractory extracranial GCTs in settings where high-dose chemotherapy and autologous stem cell transplant are not readily available.

## Introduction

Germ cell tumors (GCTs) represent a rare and heterogeneous group of neoplasms, accounting for approximately 3.5% of pediatric malignancies and a minority of solid tumors in adults [1,2]. The incidence of extracranial GCTs varies geographically, but the annual incidence is estimated to be around 0.5 cases per 100,000 children and adolescents [3]. The introduction of platinum-based chemotherapy has dramatically improved survival rates in GCTs [4]. A subset of patients either relapse after initial response or exhibit refractory disease, constituting a significant therapeutic challenge.

Relapsed (rel-GCT) and refractory (ref-GCT) extracranial GCTs represent a small but clinically significant subset, as outcomes in this group are far inferior compared to those with primary disease [5]. Approximately 20-30% of patients with advanced GCT will either not achieve remission or subsequently relapse following first-line therapy [6,7]. For these patients, salvage chemotherapy regimens remain the mainstay of treatment. Reported response rates to first-line salvage chemotherapy range between 20-50% depending on tumor location, prior therapy, and risk stratification [8].

High-dose chemotherapy (HDCT) with autologous stem cell rescue has become a standard approach for selective patients with chemosensitive relapse, improving long-term survival in selected subgroups [9,10]. Such approaches are not universally available or feasible in low- and middle-income countries (LMICs) due to resource limitations and toxicity concerns. Despite advances in initial therapy, relapsed or refractory GCTs continue to pose a significant risk for treatment failure and mortality, especially in resource-limited settings. There is a paucity of real-world data from Pakistan and the broader South Asian region regarding the efficacy of conventional salvage chemotherapy for rel/ref-GCTs, particularly in the absence of stem cell rescue. Understanding the outcomes, response rates, and prognostic factors in this population is essential for optimizing treatment protocols, counseling families, and resource allocation in LMICs. The present study evaluates remission rates and survival outcomes of pediatric relapsed/refractory GCTs managed with conventional salvage chemotherapy regimens, without the use of stem cell rescue.

## Materials and methods

This retrospective cohort study was conducted at the Shaukat Khanum Memorial Cancer Hospital and Research Centre (SKMCH&RC), Lahore, Pakistan. The study period spanned ten years, from January 2011 to December 2022. Ethical approval for the study was obtained from the Institutional Review Board of SKMCH&RC, Lahore (number EX-05-07-24-1). Owing to the retrospective nature of the study and anonymized analysis of patient data, the requirement for individual informed consent was waived by the ethics committee. No prior sample size calculation was performed due to the rarity of relapsed and refractory GCTs, and all patients fulfilling eligibility criteria were included for analysis. Inclusion criteria comprised patients aged 18 years or less with histologically confirmed extracranial GCTs who received definitive treatment initially, and later presented with relapsed or progressive GCT after having received primary chemotherapy. Exclusion criteria were the absence of treatment records or insufficient follow-up data to allow assessment of outcomes. A non-probability consecutive sampling technique was used.

Data were extracted from electronic medical records and the institutional oncology registry using a standardized data collection form. Collected variables included demographic details such as gender and age, clinical data like primary tumor site, risk stratification at diagnosis, and treatment details including type and number of chemotherapy cycles, surgical interventions, use of radiotherapy, and outcomes like relapse, progression, survival status, and date of last follow-up. At initial presentation, patients were stratified by risk: completely resected teratomas and stage I gonadal tumors were classified as low risk, stage IV ovarian and stage III-IV extragonadal GCTs as high risk, and all other cases as intermediate risk.

Prior to commencing therapy, all patients underwent a detailed baseline assessment that included physical examination, complete blood count, liver and renal function tests, serum electrolytes, and measurement of tumor markers such as alpha-fetoprotein (AFP) and beta-human chorionic gonadotropin (β-hCG). Audiological assessments were conducted prior to and during the course of platinum-based chemotherapy. Treatment response was assessed by tumor marker normalization and radiological evaluation after four to six cycles of chemotherapy, or as clinically indicated. 

Relapse was defined as the reappearance of disease after initial response, indicated by tumor marker elevation greater than five times the upper limit of normal, serially increasing tumor markers, or unequivocal clinico-radiological progression, singly or in combination. Refractory GCT was defined as progression occurring during primary treatment or recurrence within four weeks of completion of therapy, as evidenced by biochemical, clinical, or radiological criteria. Event-free survival (EFS) was defined from initiation of salvage chemotherapy after confirming relapse or progression to second relapse, progression, or death, with censoring at last follow-up for event-free patients. Overall survival (OS) was measured from initiation of salvage therapy after confirming relapse or progression to death or last follow-up.

Patients with stage I gonadal tumors managed with surgery alone received bleomycin, etoposide and cisplatin (BEP; cisplatin 20 mg/m²/day, D1-5; etoposide 100 mg/m²/day, D1-5; bleomycin 15 IU/m², D1) or carboplatin, etoposide, and bleomycin (JEB; carboplatin 560 mg/m² substituted for cisplatin). Relapsed/refractory intermediate- and high-risk patients previously treated with BEP/JEB received vinblastine, ifosfamide and cisplatin/vinblastine, ifosfamide, carboplatin (VeIP/VeIC; vinblastine 6 mg/m², D1; ifosfamide 1500 mg/m²/day, D1-5; cisplatin 20 mg/m²/day, D1-5, or carboplatin 560 mg/m², D1) or paclitaxel, ifosfamide and cisplatin (TIP; paclitaxel 135 mg/m², D1; ifosfamide 1500 mg/m²/day, D1-5; cisplatin 20 mg/m²/day, D1-5, or carboplatin 560 mg/m², D1). Irinotecan, paclitaxel, and oxaliplatin (IPO; oxaliplatin 100 mg/m², irinotecan 200 mg/m², paclitaxel 80 mg/m² weekly) were given every three weeks for 3-4 cycles. Surgery was performed at relapse or post-salvage therapy where indicated, while radiotherapy was used for inoperable disease or local control.

Data were analyzed using IBM-SPSS Statistics version 26.0 (IBM, Inc., Armonk, US). Continuous variables were summarized as means and standard deviations or medians and interquartile ranges, as appropriate, while categorical variables were described using frequencies and percentages. The Kaplan-Meier method was used to estimate EFS and OS, with comparisons between groups performed using the log-rank test. A multivariate Cox proportional hazards regression model was applied to determine independent predictors of survival outcomes. Statistical significance was set at a two-tailed p-value of less than 0.05.

## Results

In a total of 51 patients analyzed, 25 (49%) were male, and 26 (51%) were female. The median age at the time of diagnosis was 8 (3-12) years, ranging between one and 18 years. The primary site of GCTs was gonadal and extragonadal, found in 33 (64.7%) and 18 (35.3%) patients, respectively. The most common baseline histology was yolk sac, followed by mixed types, in 27 (52.9%) and 13 (25.5%) patients, respectively. Disease categories were low, intermediate, and high risk among 16 (31.4%), 11 (21.6%), and 24 (47.1%) patients, respectively. The most common first-line treatment was JEB, in 25 (49%) patients, whereas 16 (31.4%) patients were managed with surgery or observation. The distribution of risk categories at initial presentation showed a significant trend towards higher risk in extragonadal GCTs (p=0.013). First-line treatment options also showed a significant association with respect to gonadal and extragonadal GTCs (p=0.020; see Table 1).

**Table 1:** Comparison of baseline characteristics with primary site of GCTs (n=51) GCTs - germ cell tumors; JEB - carboplatin, etoposide, and bleomycin; BEP - bleomycin, etoposide and cisplatin; PIE - paclitaxel, ifosfamide, etoposide

Charactertistics		Godanal (n=33)	Extragonadal (n=18)	p-value
Gender	Male	17 (100%)	7 (38.9%)	<0.001
	Female	-	11 (61.1%)	
Age (years), median (IQR)		8.0 (2.5-12.0)	7.0 (2.8-12.3)	0.587
Risk category	Low	15 (45.5%)	1 (5.6%)	0.013
	Intermediate	6 (18.2%)	5 (27.8%)	
	High	12 (36.4%)	12 (66.7%)	
Primary metastatic sites	None	26 (78.8%)	15 (83.3%)	0.899
	One	3 (9.1%)	1 (5.6%)	
	2-3	3 (9.1%)	1 (5.6%)	
	>3	1 (3.0%)	1 (5.6%)	
Baseline histology	Embroynal	1 (3.0%)	1 (5.6%)	0.759
	Yolk sack	17 (51.5%)	10 (55.6%)	
	Mature teratoma	3 (9.1%)	-	
	Immature teratoma	2 (6.1%)	2 (11.1%)	
	Mixed	8 (24.2%)	5 (27.8%)	
	Dysferminoma	1 (3.0%)	-	
	Seminoma	1 (3.0%)	-	
First-line treatment	JEB	13 (39.4%)	12 (66.7%)	0.020
	BEP	5 (15.2%)	4 (22.2%)	
	PIE	-	1 (5.6%)	
	Observation	15 (45.5%)	1 (5.6%)	

Isolated local recurrence was observed in 18 patients (35.3%), metastatic recurrence in 13 (25.5%), and combined recurrence in 20 (39.2%). Gonadal GCTs were seen in 33 patients (64.7%), with 18 (35.2%) testicular and 15 (29.4%) ovarian primaries. Metastatic relapse predominated in testicular tumors (n=12, 70.6%), while ovarian relapses were more often local (n=8, 53.3%).Refractory GCTs occurred more frequently in extragonadal primaries (n=12, 66.6%), with sacrococcygeal (n=6, 33.3%) and mediastinal (n=3, 16.6%) sites being the most common. Yolk sac tumor remained the predominant histology across all relapsed/refractory cases. Six patients initially diagnosed with teratomas (mature, n=3; immature, n=4) relapsed with malignant yolk sac elements confirmed by raised AFP and/or histology, two were extragonadal and four ovarian. Biochemical progression preceded clinical or radiological relapse in 41 (80.3%), while 10 non-secretory GCTs relapsed on imaging or histology. Sixteen patients (31.4%) treated with surgery alone at presentation received BEP/JEB at relapse. VeIP/VeIC was the commonest salvage regimen (n=28, 54.9%), while paclitaxel, ifosfamide and cisplatin/paclitaxel, ifosfamide, and carboplatin (TIP/TIC, n=3, 5.9%) and IPO (n=1, 2%) were used in extragonadal cases. One patient with dysgerminoma received BEP salvage without events. Among 41 assessable patients, tumor marker normalization after two cycles occurred in 28 (68%) patients. Normalization was more frequent in those previously exposed to platinum (n=27, 79.4%). Local therapy was feasible in 30 patients; 20 underwent surgery at relapse or after 2-4 salvage cycles. Radiotherapy (RT) was delivered with palliative intent in seven (13.7%), and curative intent in three (5.9%) patients; six (11.8%) received local-field ​​​​RT, and four (7.8%) received metastatic-site RT.

**Figure 1:**
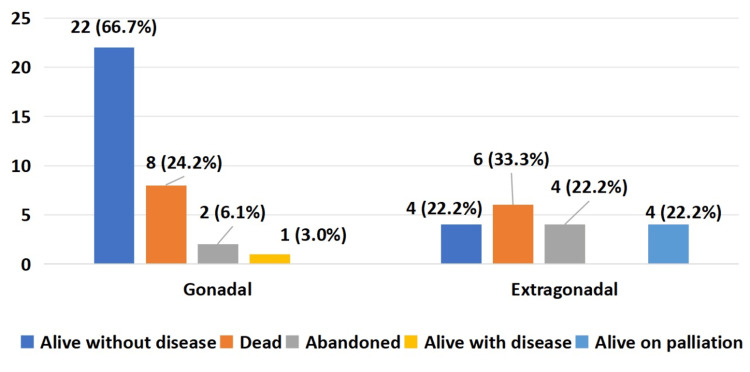
Final outcome with respect to primary site of GCTs (n=51) GCTs - germ cell tumors

At final evaluation among patients with gonadal GCTs (n=33), 22 (66.7%) were alive without disease, eight (24.2%) died, two (6.1%) had abandoned treatment, and one (5.9%) was alive with disease on follow-up. For extragonadal GCTs (n=18), four (22.2%) were alive without disease, six (33.3%) had died, four (22.2%) had abandoned treatment, and four (22.2%) were alive with disease on follow-up. In the ovarian GCT group (n=16), 10 (62.5%) were alive without disease, five (31.3%) had died, and one (6.3%) had abandoned treatment, while none were alive with disease or on palliation. Remission rates varied significantly by primary tumor site (p=0.004), and the details are depicted in Figure 1.

**Figure 2:**
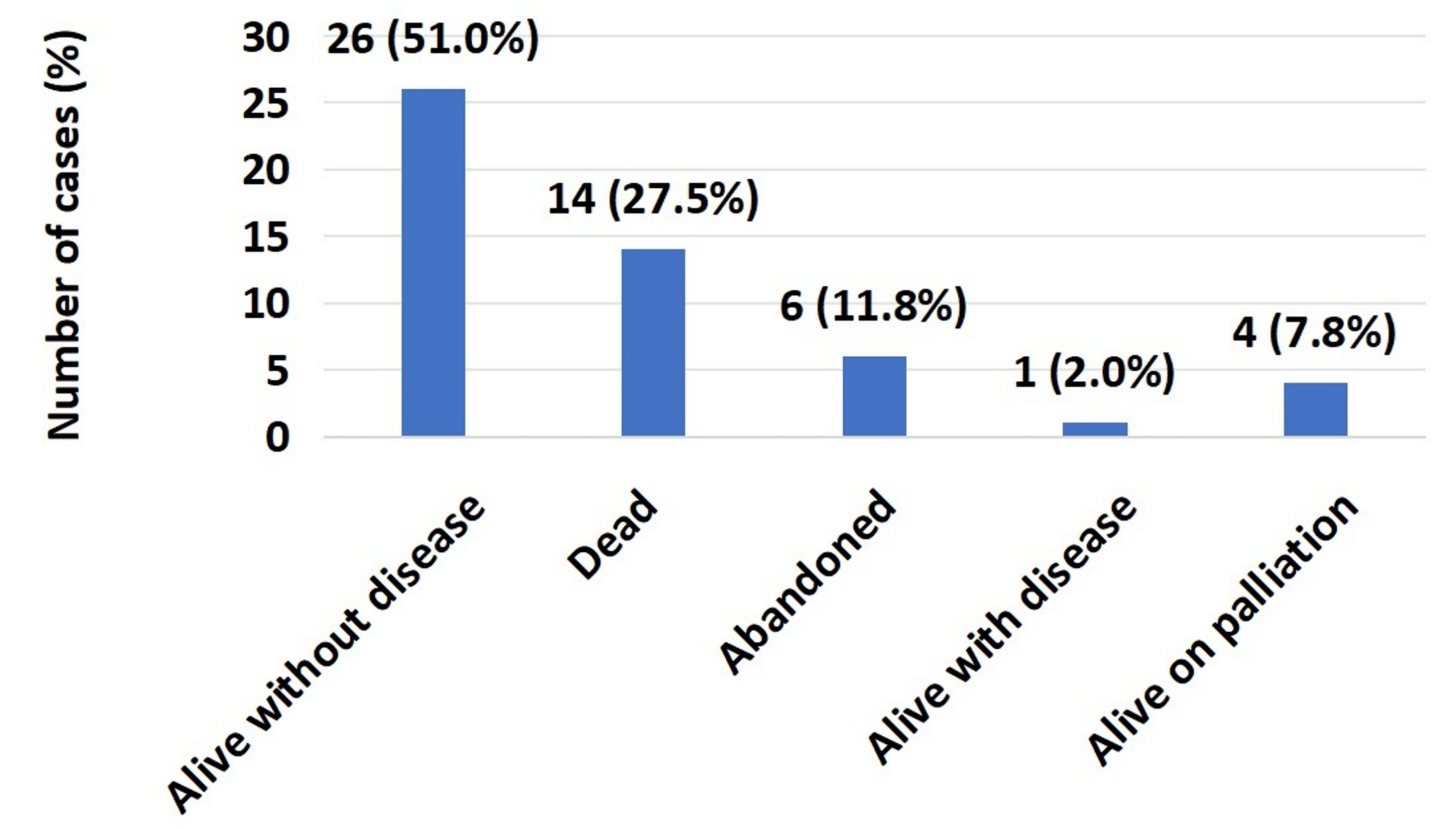
Final outcomes (n=51) Alive without disease: patients achieving complete remission and disease-free survival at last follow-up. Dead: patients who succumbed to progressive or recurrent disease. Abandoned: patients who discontinued or defaulted treatment before completion. Alive with disease: patients with persistent active malignancy at last assessment. Alive on palliation: patients receiving palliative chemotherapy or supportive care for advanced or progressive disease.

The median duration of follow-up was 32 months (IQR: 20-61), ranging between seven and 128 months. In terms of final outcomes, 26 (51%) patients were alive without disease, while 14 (27.5%) patients reported mortality (Figure 2).

**Figure 3:**
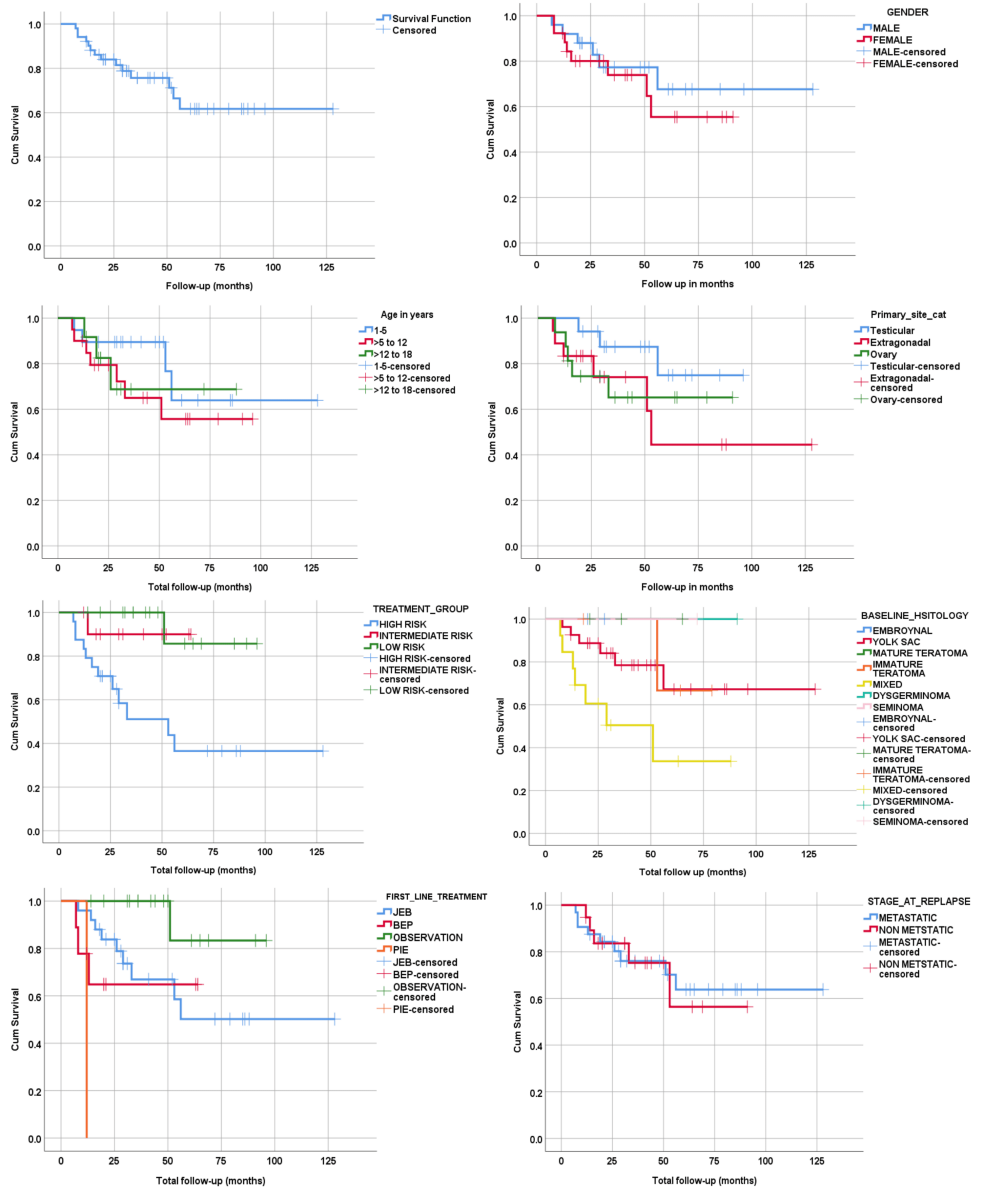
Kaplan-Meier survival curves showing overall survival GCTs - germ cell tumors; JEB - carboplatin, etoposide, and bleomycin; BEP - bleomycin, etoposide and cisplatin; PIE - paclitaxel, ifosfamide, etoposide

The mean OS was 90.9 months (95% CI: 74.7-107.1). At one, two, three, and five years, the OS was 92.2%, 84.3%, 78.4%, and 72.5%, respectively. Comparison of OS by gender (p=0.530), age groups (p=0.561), primary site of GCTs (p=0.315), baseline history (p=0.152), and stage of relapse (p=0.935) did not exhibit any significant differences, while risk category (p=0.004) and first-line treatment options (p=0.001) varied significantly (see Figure 3).

**Figure 4:**
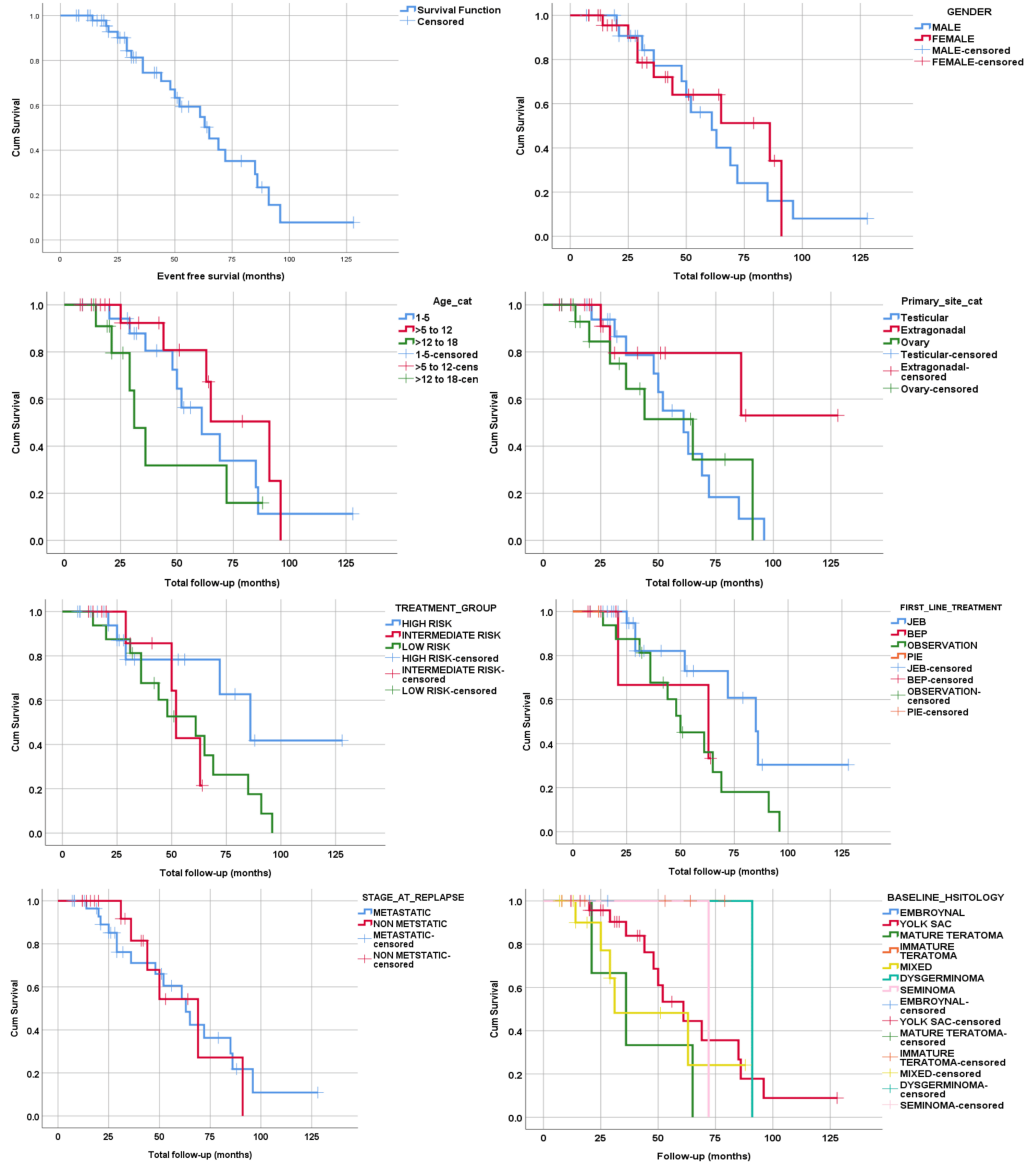
Kaplan-Meier curve showing event free survivals GCTs - germ cell tumors; JEB - carboplatin, etoposide, and bleomycin; BEP - bleomycin, etoposide and cisplatin; PIE - paclitaxel, ifosfamide, etoposide

The median duration of EFS was 65 months (95% CI: 47.8-82.2). The EFS at one, two, three, and five years was 90.2%, 72.5%, 60.8%, and 50%, respectively. Gender (p=0.657), age (p=0.218), primary site of GCTs (p=0.172), baseline history (p=0.332), disease risk category (p=0.185), first-line treatment option (p=0.209), and stage at relapse (p=0.935) did not vary significantly with respect to EFS (see Figure 4).

In the multivariate Cox proportional hazards regression analysis, risk category at relapse was independently associated with mortality among patients with relapsed or refractory extracranial GCTs. Compared to the low-risk group, patients in the high-risk group had a significantly higher hazard of death (hazard ratio, HR: 12.27, 95% CI 1.51-99.6; p=0.019). The intermediate-risk group also had an increased hazard compared to low-risk (HR: 1.80, 95% CI: 0.11-29.45), but this was not statistically significant (p=0.679). No statistically significant associations with mortality were observed for gender (female vs male HR: 0.72, 95% CI: 0.14-3.62; p=0.690), age group (5-12 years vs 1-5 years HR: 2.30, 95% CI: 0.49-10.91; p=0.293; 12-18 years vs 1-5 years HR: 0.89, 95% CI: 0.19-4.25; p=0.882), or primary tumor site (testicular vs extragonadal HR: 1.07, 95% CI: 0.20-5.80; p=0.937; ovary vs extragonadal HR: 0.57, 95% CI: 0.11-2.99; p=0.507), as shown in Table 2.

**Table 2:** Multivariate Cox regression analysis for mortality in relapsed/refractory extracranial GCTs GCTs - germ cell tumors

Predictors		p-value	Hazard ratio	95% CI
Gender	Male	Reference		
	Female	0.69	0.72	0.14-3.62
Age groups (years)	1-5	Reference		
	>5 to 12	0.293	2.30	0.49-10.91
	>12 to 18	0.882	0.89	0.19-4.25
Primary site of GCTs	Testicular	0.937	1.07	0.20-5.80
	Ovary	0.507	0.57	0.11-2.99
	Extragonadal	Reference		
Disease category	Low risk	Reference		
	Intermediate risk	0.679	1.80	0.11-29.45
	High risk	0.019	12.27	1.51-99.60

## Discussion

This study reported that conventional salvage chemotherapy, in the absence of stem cell rescue, resulted in modest long-term survival and remission rates for children with relapsed or refractory extracranial GCTs treated in a tertiary care setting in Pakistan. The five-year OS in this cohort reached 72.5%, and EFS at five years was 50%. Remission rates and survival outcomes observed in the present cohort correspond to, yet slightly exceed, the survival rates reported by Ramanathan et al., who studied children with relapsed or refractory extracranial GCTs treated with conventional salvage chemotherapy without stem cell support [11]. That study demonstrated a five-year OS of 50% and EFS of 42.4%, while the present study achieved a 5-year OS of 72.5% and EFS of 50%. The difference may be attributed to variations in patient selection, intensity of supportive care, or differences in follow-up duration. The median follow-up in this study was relatively shorter (32 months) compared to the 60 months reported by Ramanathan et al., which could have influenced survival estimates [11]. There is also potential influence from differing proportions of risk stratification and primary tumor sites at baseline. Briones et al., synthesizing evidence from prospective trials, found no significant OS benefit for high-dose chemotherapy with autologous stem cell transplant (ASCT) compared to conventional-dose chemotherapy, with a hazard ratio for survival of 0.98 and overlapping confidence intervals. Non-randomized trials included in their analysis suggested that repeated cycles of high-dose chemotherapy (HDCT) may yield superior responses, especially in those who achieve disease control prior to transplant [12]. Villela et al. reported five-year OS and EFS of 47.1% and 44.1%, respectively, for pediatric patients with extracranial GCTs receiving HDCT/ASCT [13]. The present study, while employing only conventional salvage regimens, demonstrated survival rates comparable to or exceeding those in pediatric transplantation series, though with a patient population that included a higher proportion of gonadal primaries and fewer high-risk extragonadal tumors.

This study also underscores the heterogeneity in outcomes according to disease site and risk group. Patients with testicular primaries had the highest rates of being alive without disease (70.6%), and those with extragonadal primaries had the lowest (22.2%). These differences are mirrored in the report by Topal et al., which concluded that extragonadal GCTs have a more unfavourable prognosis compared to gonadal GCTs, even after HDCT and ASCT. Their cohort, which included adult patients undergoing HDCT/ASCT for extragonadal GCT, experienced a median OS of 12.2 months, which is substantially lower than the 90.9 months observed in the current study [14]. The differences in patient age, intensity of prior therapy, and extent of metastatic disease at relapse likely explain the disparity in survival.

The significant prognostic impact of disease risk category at relapse was evident in this study, where high-risk disease was associated with a more than twelvefold increase in the hazard of mortality compared with low-risk disease (HR: 12.27, 95% CI: 1.51-99.60, p=0.019). This echoes the findings of Ramanathan et al., who identified much poorer outcomes in high-risk and platinum-exposed patients, with a five-year EFS of only 6.5% for high-risk disease [11]. The close linkage between risk group and prognosis is also observed in the series by de Pasquale et al., who noted an overall survival of 54.5% for platinum-refractory patients compared to 91.5% for the relapsed group, despite a salvage treatment strategy that incorporated both standard and high-dose regimens [15].

The response to salvage chemotherapy was closely linked to tumor marker normalization after two cycles. In the present study, 68% achieved normalization, and these patients constituted the majority of long-term survivors. The relationship between early marker response and outcome is well documented. Tiyaamornwong et al. demonstrated that post-surgical AFP normalization within one month was associated with improved five-year OS (93.4% vs 69.4%, p=0.031) [16]. The current findings are consistent with these results and support the use of tumor marker dynamics as a surrogate for early treatment response and long-term prognosis. Patterns of relapse further inform treatment outcomes [17,18]. This cohort saw isolated local recurrence in 35.3% of cases, metastatic recurrence in 25.5%, and combined recurrence in 39.2%. Metastatic disease at relapse, particularly with extragonadal primaries, was associated with poor prognosis. This pattern aligns with the work of Ramanathan et al., where local relapses had superior five-year EFS (64%) compared with metastatic (23%) or combined relapses (0%) [11].

The choice of salvage chemotherapy regimens varied in the present cohort, with VeIP/VeIC being the most frequently used (54.9%), while a minority received TIP/TIC or IPO. The efficacy of these regimens is consistent with the published literature that endorses platinum, etoposide, ifosfamide, and paclitaxel-based combinations as standards for salvage therapy in relapsed GCTs [19,20]. Pfister et al. highlight that, while conventional cisplatin-based chemotherapy remains standard, retrospective data suggest that sequential high-dose chemotherapy may improve survival by 10-15% for relapsed patients, although robust prospective evidence remains lacking for the pediatric population [21]. Comparative analysis with HDCT/ASCT series in children by Giorgi et al. reveals that salvage high-dose chemotherapy can achieve long-term remissions in up to half of children with extragonadal GCTs, with a plateau in two-year progression-free survival at 50% [22]. The absence of a high-dose chemotherapy or transplant option in the present study may have constrained EFS and OS, particularly in the high-risk and extragonadal groups, supporting the rationale for exploring these modalities prospectively in similar populations.

Clinical implications of these findings are significant for practice in resource-limited settings. The observed survival rates indicate that conventional salvage chemotherapy, supported by multidisciplinary care, can achieve meaningful outcomes even without access to stem cell transplantation [23,24]. The substantially lower survival for high-risk and extragonadal cases points to the need for treatment intensification or novel therapeutic strategies in these subgroups. Incorporation of high-dose chemotherapy, improved access to ASCT, and early identification of poor responders by tumor marker dynamics could further enhance survival rates.

The current study carries several limitations. The retrospective design introduces the potential for selection bias and incomplete data capture. The relatively small sample size, a consequence of the rarity of relapsed/refractory pediatric GCTs, limits statistical power for subgroup analysis and may obscure subtle prognostic effects. Shorter median follow-up compared to some published series could inflate survival estimates, as late relapses may not yet have occurred. These limitations could be addressed in future research through multicentre collaboration to increase sample size, adoption of prospective data collection to ensure completeness and accuracy, and longer follow-up to capture late events.

## Conclusions

Salvage chemotherapy regimens provide an effective option for children with relapsed or refractory extracranial GCTs in settings where HDCT and ASCT are not readily available. Long-term survival is achievable, especially in patients with gonadal primaries and low-risk features. High-risk and extragonadal disease remain associated with poor outcomes, highlighting the urgent need for new therapeutic strategies, improved supportive care, and integration of novel treatment modalities in pediatric oncology programmes.
